# LiReD: A Light-Weight Real-Time Fault Detection System for Edge Computing Using LSTM Recurrent Neural Networks

**DOI:** 10.3390/s18072110

**Published:** 2018-06-30

**Authors:** Donghyun Park, Seulgi Kim, Yelin An, Jae-Yoon Jung

**Affiliations:** Department of Industrial and Management Systems Engineering, Kyung Hee University, 1732, Deogyeong-daero, Giheung-gu, Yongin-si 446-701, Korea; pdh@khu.ac.kr (D.P.); nysg6190@khu.ac.kr (S.K.); ylan@khu.ac.kr (Y.A.)

**Keywords:** data-driven fault detection, prognostics and heath management, edge computing, real-time monitoring

## Abstract

Monitoring the status of the facilities and detecting any faults are considered an important technology in a smart factory. Although the faults of machine can be analyzed in real time using collected data, it requires a large amount of computing resources to handle the massive data. A cloud server can be used to analyze the collected data, but it is more efficient to adopt the edge computing concept that employs edge devices located close to the facilities. Edge devices can improve data processing and analysis speed and reduce network costs. In this paper, an edge device capable of collecting, processing, storing and analyzing data is constructed by using a single-board computer and a sensor. And, a fault detection model for machine is developed based on the long short-term memory (LSTM) recurrent neural networks. The proposed system called LiReD was implemented for an industrial robot manipulator and the LSTM-based fault detection model showed the best performance among six fault detection models.

## 1. Introduction

The recent development of information and communication technologies has engendered the concept of the smart factory, which is expected to change the manufacturing paradigm. To implement smart factories, various technologies such as sensors, networks, cloud computing, and robots are required to make production activities adaptable and flexible for increasing productivity [1].

Condition monitoring of factory facilities and parts therein, and anticipating faults to address failures in advance, can greatly improve the reliability and productivity of the whole manufacturing process. Those subjects have been actively studied in recent years in the field of prognostics and health management (PHM), which can reduce the downtime of production lines and costs for production and break-down maintenance [2]. The main functions of PHM are diagnosis of facility faults, identification of the failure causes, prediction of the remaining useful life of the facility, and determination of the timing and methods of preventive maintenance [3].

Studies for detecting and diagnosing faults of industrial facilities have been mainly conducted since the 1980s. They have been performed primarily based on mathematical or physical-based approaches [4,5,6,7,8,9,10]. They have shown good performance in monitoring and predicting faults of industrial machines in specific conditions and environments. However, the approaches are sensitive to noise and system complexity in the actual production field. Moreover, it is difficult to determine lots of their model parameters [2].

To overcome these limitations, data-driven approaches using machine learning or artificial intelligence techniques have been proposed, which analyze a large amount of data to develop the best models. The models find hidden patterns from the historical data of facilities to identify expected faults in real time and predict future failures. If the amount of historical data is not sufficient, the data-driven models may not perform well compared to physical-based models [11]. However, owing to technological advancements in various fields, the performance of the data-driven model has steadily improved and is expected to further advance. Furthermore, modern systems are becoming increasingly complex. Thus, the development of mathematical or physical-based models is also requiring more endeavor and prior knowledge. On the other hand, the data-driven models can be developed to adequately approximate real systems based on the huge collected data.

Meanwhile, with recent advancements in cloud computing technology, many enterprises can have large storage and sufficient computing capacity by using cloud services instead of depending on their own physical infrastructure. Cloud services help reduce the costs of investing in data centers or servers, and its users can flexibly control the use of computing resources. However, limitations exist in cloud computing. When a large amount of data is transmitted to a single cloud center and processed there, a bottleneck problem occurs. It results in a delay in transmitting, processing, and receiving data. Such a problem is a fatal drawback in a smart factory that must perform massive analyses in real time. In addition, with the advent of the industrial internet of things (IIoT), the number of sensors attached to the facilities and the amount of data collected from them is expected to exponentially grow. This means that an increasing amount of cloud computing resources is needed, which will increase the network costs.

To overcome this problem, the concept of edge computing can be used. Edge computing is the technology that makes it possible to quickly perform the necessary computational tasks in the network edge, i.e., the middle of the data producer and the cloud center [12]. By performing the necessary computing tasks close to the machine that produces the data, the workload that is concentrated in the central cloud can be reduced. Additionally, for simple tasks that do not require communication with a central cloud center, such as simple operations of facilities, it is more cost-effective to process and analyze data without network communications.

In this paper, we propose the light-weight real-time fault detection system (LiReD) for edge computing. The system employs a long short-term memory recurrent neural network (LSTM)-based deep learning model for fault detection as well as an edge device based on an edge computing concept to overcome the limitations mentioned above. The edge device can collect data in real time generated from facilities using a single-board computer and a sensor. At the same time, the developed deep learning model can detect machine faults by using the gathered data. Additionally, a monitoring module is included to visualize the state of the machine. The proposed architecture was implemented for an actual industrial facility to demonstrate its performance and effectiveness. The contributions of this paper are outlined below:

- *Lightweight system for edge computing*—The proposed system is based on the edge computing concept and can detect faults by collecting and analyzing data directly from facilities. It consists of a simple single-board computer, a sensor, and open-source software. Thus, it can be quickly and easily configured.
- *Real-time fault detection based on machine learning*—The proposed system adopted the machine learning approach which learns the collected data and improves the prediction performance. The developed model based on machine learning is loaded on an edge device to detect faults in real time.
- *Implementation and evaluation for the industrial robot manipulator*—The proposed system was validated on an actual industrial robot manipulator. In addition, the developed LSTM-based model showed the best performance among several models for the data collected from the manipulator.

The remainder of this paper is structured as follows: Section 2 overviews related research. In Section 3, the overall architecture of the proposed LiReD system and each component are explained. Section 4 describes the application of this study to actual systems. Section 5 presents the experimental results using the LSTM model developed for fault detection. Finally, Section 6 presents our conclusions and future work.

## 2. Related Work

### 2.1. Data-Driven Approaches for Fault Detection

Yang et al. [13] presented a methodology of machine fault diagnosis using random forest, a machine learning model. Results showed that it is possible to diagnose a machine fault with fast execution speed and high accuracy of the model. Muralidharan and Sugumaran [14] compared discrete wavelet transform (DWT)-based wavelet analysis with the naïve Bayes classifier and the Bayes net classifier to diagnose the failure of a monoblock centrifugal pump. Using the proposed feature extraction method and classifier, it can be a good candidate application for fault diagnosis of the pump. Soualhi et al. [15] performed a study on fault detection and residual useful life prediction using the Hilbert–Huang transform (HHT), support vector machine (SVM), and support vector regression (SVR). HHT is one of several methods used to extract the health indicators for fault detection. It removes the noise signals and the unnecessary signals from the original vibration signals. SVM [16] is known as a supervised classification model with good performance, and the study in [16] detected the deterioration state of bearings. SVR [17] is a regression version of SVM and is used to predict bearings’ remaining useful life.

Recently, with the advancement of deep learning technology, studies using artificial neural networks have been conducted in many fields. In the field of PHM, artificial neural networks have been studied previously [18,19,20,21,22,23]. These studies were mainly based on the multi-layer perceptron (MLP), which stacks several layers of hidden layers. In [22], time-domain features were extracted from bearing vibration signals and used as inputs for artificial neural networks. The hidden layer consists of two layers, and the model is trained by the backpropagation algorithm. The proposed methodology is advantageous in that it can perform fault diagnosis by fast training of the model using simple data preprocessing and only a few inputs.

In [23], the authors proposed a method that enables the final classifier neural network to perform fault diagnosis by sequential execution of a pre-processor network and a compressor network. The compressor network uses models called recirculation networks in [23], which is identical to the neural network structure that is now known as an auto-encoder. The authors of [23] proposed a robust and good performance model by adopting a methodology to generate feature vectors representing low-frequency and high-frequency regions by encoding vibration signals and using them as inputs to the classifier.

More recently, deep learning techniques such as auto-encoder, convolutional neural networks (CNNs), and recurrent neural networks (RNNs), have been used in the PHM field in MLP [24,25,26,27,28,29]. Particularly, in the case of RNNs, most of the data used for PHM are time-series data that records changes in mechanical conditions, such as vibration, temperature, or pressure of the machine over time. Gugulothu et al. [30] proposed ‘Embed-RUL’ methodology for remaining useful life (RUL) estimation. They embedded time series data using RNNs as an encoder and drew a health index (HI) curve with it. Then, the remaining useful life is predicted by comparing the latter curve with the normal HI curve. That methodology is a useful approach when sensor data have noise and missing values, or when there is insufficient prior knowledge of machine degradation trends.

Meanwhile, RNNs is a useful model for analyzing time series data. However, if the length of the time series is long, it cannot reflect past information well. To overcome this issue, the LSTMs algorithm was applied to control the use of cell state information by employing a forget gate [31]. Yuan et al. [32] proposed a fault diagnosis and residual useful life prediction method of an aircraft engine using LSTMs. Similar to the method proposed in [30], in [33], a reconstruction model was constructed using LSTMs as an encoder and a decoder. Then, the health index was estimated using the LSTMs as an encoder and decoder, and the remaining useful life of the machine was predicted. Zhao et al. [34] proposed a tool-wearing condition-measurement method for a high-speed computer numerical control (CNC) machine simultaneously using LSTMs and CNNs. They used CNNs as a local feature extractor for time-series data and bi-directional LSTMs as a tool to predict wear. Using bi-directional LSTMs rather than basic LSTMs, they used strong temporal dependency characteristics of time series data by employing both past and future information.

### 2.2. Edge and Fog Computing

Shi et al. [12] defined the “edge” of edge computing as “any computing and network resource along the path between data sources and cloud data centers.” They introduced the effects of edge computing. Specifically, compared to traditional cloud computing, it can reduce power consumption by eliminating the use of central data centers and speed up the analysis tasks [35,36,37]. They also suggested some cases where edge computing can be applied. Those cases are described below.

When customers engage in mobile shopping, they place the objects of consumption in/out of their shopping carts. At the same time, a time delay occurs, depending on the speed of the network and the workload of the cloud server. If edge computing is used in this situation, it can solve the time delay problems and provide a good user experience. Another example is the smart home. In a smart home, it is difficult to communicate with the cloud center every time because of the large amount of data that has been generated from various devices in the home [12]. Furthermore, with respect to concerns about leakage of personal data from the home, edge computing reduces the personal privacy problem.

Wu et al. [38] proposed process monitoring and a prognosis framework based on fog computing. A sensing node was constructed using Arduino and sensors. The collected data were presented to a gateway using ZigBee communication. After processing and analyzing the data at the gateway, the system was configured to check the state of the facility from different interfaces, such as smart phones or laptops, through an edge cloud. In addition, it used the random forest model to diagnose the faults.

## 3. LiReD: Light-Weight Real-Time Fault Detection System

**Figure 1:**
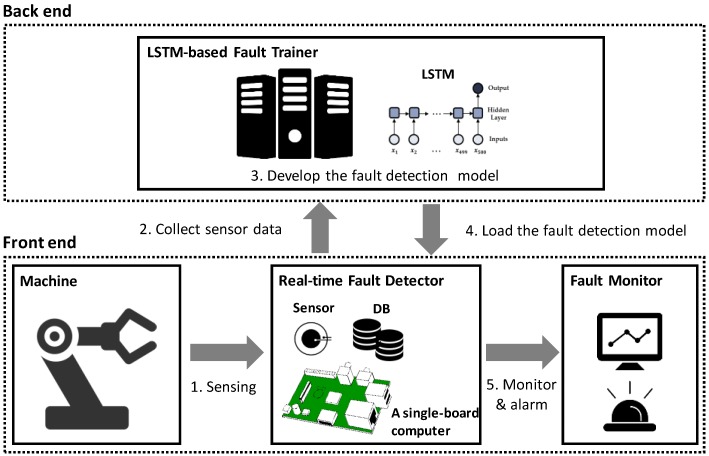
Architecture of the LiReD system.

In this section, the overall architecture of the proposed LiReD system is described. It consists of two main parts, shown in Figure 1. The front end monitors the condition of facilities and processes a fault detection in real time, while the back end performs the development of a fault detection model that will be used in the front end.

The front end includes a machine in the factory, a real-time fault detector, and a fault monitor. The *real-time fault detector* works with data from the sensor on the *machine* during its operation. The results of fault detection from the fault detector are transmitted to the *fault monitor*, which visualizes the condition of the machine and turns on the warning light for the operator.

The back end takes data collected from the real-time fault detector to develop the best model for fault detection. This developed algorithm is built into the real-time fault detector as a model. Then, the real-time fault detector uses this model to detect faults without data communication to the main server each time. In other words, it becomes an edge device that can independently collect, process, store, and even analyze data without the multiple network communication required for data analysis.

### 3.1. Real-Time Fault Detector

The real-time fault detector that is attached to the machine acts as an edge device in the LiReD system. The sensors on the detector may collect various real-time data such as vibration, temperature, and pressure during the operation of the machine. To implement the light-weight detector, the single-board computer can be used, which collect, pre-process and analyze the real-time data. Typical single-board computers include Raspberry Pi [39], Banana Pi [40], and BeagleBone [41]. In this detector, the database is configured to enable immediate storage of collected data and analysis results. The data stored in the database can be used when the fault detection models are trained in the back end.

### 3.2. LSTM-Based Fault Trainer

The fault trainer in the back end performs the development of the fault detection model. It employs the machine learning approach to find the best model of classifying normal and fault states of the machine based on the historical data collected in the front end. In the LiReD system, LSTM was adopted for the classification algorithm for fault detection.

LSTM, which improves typical RNN, is often used to address time-series data, such as speech recognition [42] or natural language processing [43]. LSTM can overcome the gradient vanishing problem, which means that the weights of the neural networks are not updated well in the long time-series data, by configuring the forget gate, input gate, and output gate to adjust the amount of information within a cell [31,44]. The forget gate determines how much information from the previous cell should be forgotten. The input gate determines how much information the current input value will receive, while the output gate decides how much information should be transmitted from the cell. Using these three gates, a good output can be created by storing the useful information for a long time and forgetting the useless information [45].

To develop good LSTM models, the appropriate hyperparameters should be found. For the fault detection LSTM modeling, hyperparameters such as the dimensionality of hidden states, the type of the optimizer, the degree of kernel regularization, and the dropout rate need to be determined. The first two parameters are important in training the LSTM models properly, while the last two affect the avoidance of overfitting.

The dimensionality of hidden states means the size of the shared weight matrix, and so it should be set upon consideration of the training data size. The optimizer is used for finding the global optimum of the loss function. RMSprop, AdaGrad, and Adam optimizers can be considered the candidates of the proper optimizer. To prevent falling into a local optimum, adjusting the learning rate and checking the trend of error decrease during training process are required.

Meanwhile, overfitting is a problem that a big neural network model often faces. To avoid the overfitting, the weight decay technique that gives penalties correspond to the big weights, which is called regularization, is useful. It is necessary to find the appropriate degree of regularization. The dropout method, which is also well known to prevent the overfitting problem, lets some weights ignored randomly according to the dropout rate in the training process. The method can improve the representation of training data as well as avoiding overfitting.

Due to the characteristics of the training data and the variety of neural network structures, finding the optimal hyperparameters is time consuming. Thus, it is generally possible to determine the combinations of suitable hyperparameters by using grid search or random search techniques. In this research, the grid search technique is used to find the best hyperparameters.

To train the LSTM-based fault detection model, the time-series sensor data with fixed length and the binary data of machine states (i.e., normal or fault states) are fed to the input nodes and the single output node of the LSTM models, respectively. In this research, the whole sequential vibration data of each operation is given to the input vector, and the machine state of the operation is used as the desired output value. The sigmoid function can be used as the activation function of a single output node to determine the binary machine state, and a binary cross-entropy function was used as the loss function to update the weights of the LSTM model based on the error between the predicted and the desired output values.

### 3.3. Fault Monitor

The fault monitor enables the operators to visually check the condition of the machine and the likelihood of a fault in the factory. Information about the facility from the real-time fault detector is visually provided in two ways. One is the dashboard of visualizing the facility changes in real time as charts, thus enabling users to view the condition trend of the facility with respect to the state of the facility just before the fault occurred. This plays an important role in a maintenance perspective by helping personnel to quickly analyze the cause of fault detection. The other way is visual and audible indication. If the probability of a facility fault derived from the analysis exceeds the threshold set by the manager, it can be visually and audibly indicated by LED light bulbs and buzzer for operators to quickly detect a fault hazard situation.

## 4. System Implementation

The LiReD system is applied to an industrial robot manipulator, which is widely used in real factories. The robot manipulator is a facility used for the grinding process. The tip of the manipulator absorbs the processed product so that it can perform two consecutive jobs. It requires approximately about 50 s to process the absorbed product.

**Figure 2:**
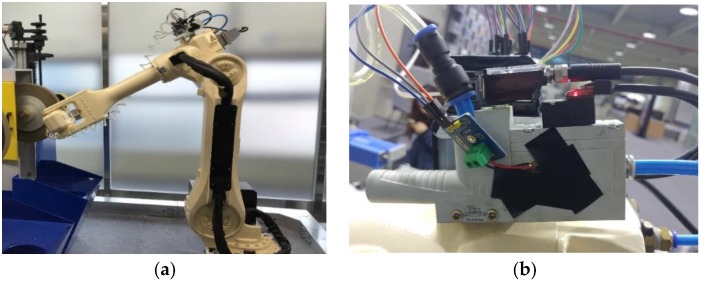
Target facility for fault detection: (**a**) grinding robot manipulator; (**b**) vacuum ejector.

In the implementation for this study, the manipulator fault was defined to drop the processed product during the process. The cause of the fault was found in the vacuum ejector mounted on the manipulator. The vacuum ejector causes the product to be absorbed to the manipulator. If the high-pressure air is inflated in the ejector, the manipulator tip becomes a vacuum. Then, the processed product is fixed to the manipulator tip. If there is a gap in the ejector body or a hole in the hose to which the air is injected, the work-piece will be dropped during the process. Figure 2 presents photographs of the manipulators and vacuum ejector used in this study implementation.

### 4.1. Real-Time Fault Detector

With consideration of cost, performance, and availability of ample development references, Raspberry Pi was selected as the main board of the real-time fault detector, which is an edge device. Raspberry Pi is a small computer that is approximately 8 cm wide, 5 cm long, and 1 cm thick. It has USB, LAN, HDMI, audio, and video ports for various input and output operations. In addition, general-purpose input-output (GPIO) connectors exist that enable additional devices, such as sensors and LED bulbs, to be connected to the board [46]. The specifications for the Raspberry Pi unit used in this study are outlined as follows [39,47]:

- Model: Raspberry Pi 3 Model B
- CPU: Quad Core 1.2 GHz Broadcom BCM 2837 64 bit
- Memory: 1 GB
- Storage: 16 GB
- Size: 85.60 mm × 56.5 mm × 17 mm
- Weight: 45 g
- On-board network: BCM 43438 wireless LAN and Bluetooth Low Energy (BLE)
- Power ratings: 300 mA (1.5 W) average when idle, 1.34 A (6.7 W) maximum under stress (monitor, keyboard, mouse and WiFi connected)

In this study, the piezoelectric accelerometer was used to collect the vibration signal from the vacuum ejector to diagnose the condition of the machine. The piezoelectric accelerometer is a sensor that uses the piezoelectric effect and outputs the magnitude of force applied to the sensor at voltage. A sensor was attached to the side of the vacuum ejector to measure the vibration signal produced while the device was operating. The specifications of the piezoelectric accelerometer are as follows [48].

- Model: Ceramic Piezo Vibration Sensor
- Working voltage: 3.3 V or 5 V
- Working current: <1 mA
- Interface: Analog
- Size : 30 mm × 23 mm
- Weight: 5 g

This sensor has an analog interface type. However, since the GPIO of Raspberry Pi only allows digital signal inputs, an analog to digital (A/D) converter was used to convert the analog vibration signal into a digital signal for transmission to Raspberry Pi. The used converter was the MCP 3008 model of Microchip [49].

MongoDB, a type of NoSQL database, was used for storing the facility data and analysis results. MongoDB stores data in the form of a document, making it easy to change the structure of the data, and it was configured in Python, Java, and C++. This enabled rapid application development, including the database [50].

In this study, Python’s PyMongo library [51] was used to construct the database. It is available for inserting and reading vibration signal values to and from the database at regular intervals. Any user who can handle Python can perform data storage and reading operations without SQL statements or knowledge of the data structure of relational databases.

### 4.2. LSTM-Based Fault Trainer

The fault detection algorithm was developed using Keras, Python deep learning library. Keras is a high-level neural network API that works on the basis of TensorFlow, Microsoft congnitive toolkit (CNTK), or Theano [52]. Simple and intuitive code usage makes it easy to construct neural networks models.

In this research, to find the optimal hyperparameters, the grid search approach was applied. The dimensionality of the hidden state was set to 50 among 25, 50, 100 and 200. The hyperbolic tangent function was chosen between the rectified linear unit (ReLU) and the hyperbolic tangent function, while a sigmoid function was used for a single output node to perform binary classification. In addition, Adam optimizer was selected among RMSProp, Adam and Adadelta optimizers. For L2 regularization, the lambda for kernel regularization was set to 0.01 among 0.001, 0.01, and 0.1. The He-normal initializer was chosen between the He-normal initializer and the Glorot-normal initializer. The dropout rate of the model was set to 0.3 among 0.2, 0.3, 0.4, and 0.5. Finally, the mini-batch size of 32 was selected between 32 and 64. 

**Figure 3:**
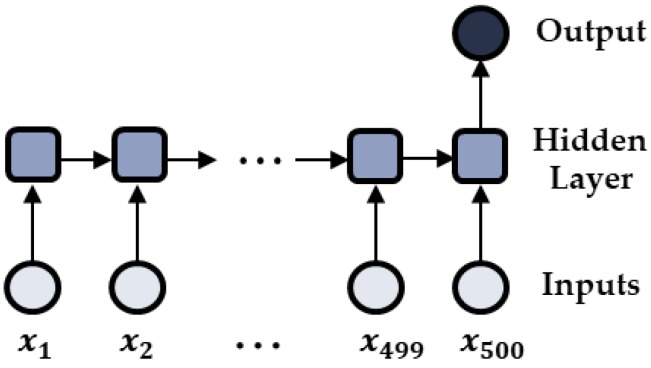
Architecture of the developed LSTM model.

The best hyperparameters were selected to minimize the training errors through 5-fold cross validation. In this work, the training errors were calculated with the loss function of the cross-entropy function. The early stopping technique was also applied to avoid the overfitting of the LSTM models. In other words, in the process of training, if the training error does not decrease for five epochs any more, the training stops. The model that was saved at the five epochs before stopping is selected as the best model. The structure of the developed LSTM model is shown in Figure 3.

### 4.3. Fault Monitor

A Node-Red dashboard was used in the fault monitor in the LiReD system to display the analysis results from the real-time fault detector. Node-Red is a flow-based programming tool that runs on Node.js. It is easy to implement various functions in JavaScript with it, and it offers rich embedded libraries [53,54,55].

The dashboard is one of the Node-RED libraries. And it can visualize data quickly and easily. Moreover, it can display real-time changes in data [56]. When configuring the database, the local server is opened in the fault detector. Thus, if users know the IP address and port number of the local server, they can view the dashboard from other display devices without having to be wired.

**Figure 4:**
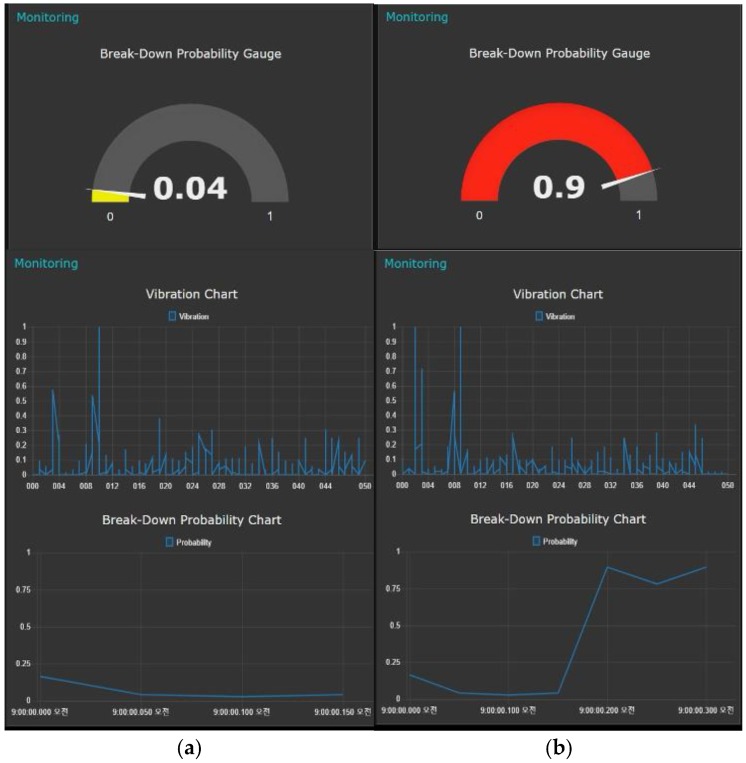
Dashboard of the fault monitor: (**a**) normal states; (**b**) fault states.

If the probability of the facility’s fault derived from the fault detection model exceeds the threshold set by the user, the LED bulb gives warning lights. The result of the configured fault monitor is shown in Figure 4.

## 5. Experiments

### 5.1. Data

**Figure 5:**
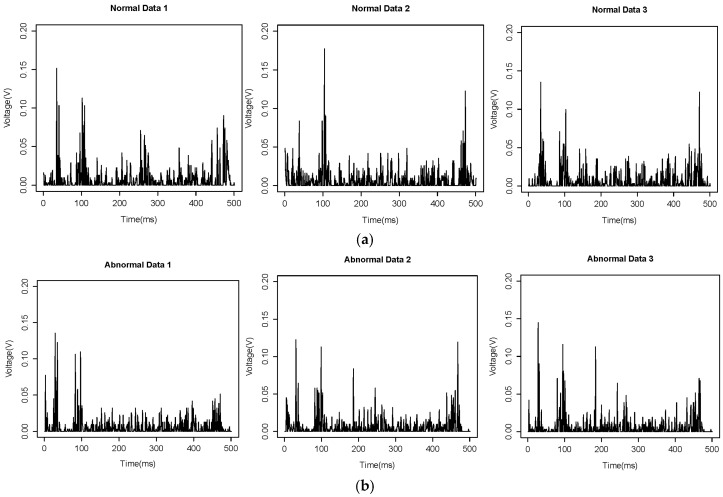
Vibration sensor: (**a**) normal state; (**b**) fault state.

To develop a fault detection model of the facility, normal state data and fault state data are required. To collect data on fault situations in this study, the fault situations of the facility were arbitrarily generated. This means that the amount of air injected into the vacuum ejector was adjusted to drop the processed product. The vibration signal collected during this process is defined as fault data. All data were collected at 0.1 second intervals to gather approximately 500 vibration signals per cycle of the process. Sixty normal-state data and fifty fault-state data were collected and used to develop the fault detection algorithms. Figure 5 shows the collected normal state data and fault state data.

### 5.2. Baseline Algorithms

To verify the developed LSTM-based model, k-NN + DTW (k = 1, 3, 5), random forest (RF), and support vector machine (SVM) were used. k-NN + DTW is known to perform well in time-series data classification, while RF and SVM are also known as good-performance algorithms in the machine learning field [57].

The k-nearest neighbor (k-NN) algorithm is a simple algorithm for classification based on the distance similarity between data. It identifies k data classes closest to the target data. For example, if k = 3, the k-NN algorithm identifies and groups the classes of the three nearest data (top-three nearest neighbors) from the classification target. In particular, the dynamic time warping (DTW) method is usually used as a measurement of distance between two time-series data [58]. DTW can effectively compare time-series data with similar shapes, but with some variation in time axes.

RF is a tree-based ensemble algorithm that is frequently used in the field of machine learning. It creates several trees that perform the classification and adopt the classification results derived from each tree using a voting method. In particular, since only a few features contained in the data are used to create one tree, it results in a higher diversity model that is not over-fitted to the data.

SVM uses the kernel to transform the data distributed in multiple dimensions to different dimensions and then finds the hyperplane that most effectively divides the data. SVM is known to perform well for high-dimensional data if they have appropriate hyper-parameters. A detailed explanation can be found in the literature [59,60].

For the RF and SVM algorithms, the time domain features that represent the data characteristics were extracted as shown in Table 1, rather than using the raw data directly as input [61,62]. To find the best SVM model, the sigmoid function was chosen as the SVM kernel among RBF, polynomial, sigmoid, and linear functions, the regularization parameter *C* was set to 50 among 0.01, 0.1, 1, 10, 25, 50, 100 and 1000, and the Kernel coefficient gamma was set to 0.01 among 0.1, 0.01, 0.001, 0.0001 and 0.00001. All the parameters were determined through 5-fold cross validation for the training dataset, and the detail of the applied cross validation technique is introduced in the next subsection.

### 5.3. Performance Evaluation

**Figure 6:**
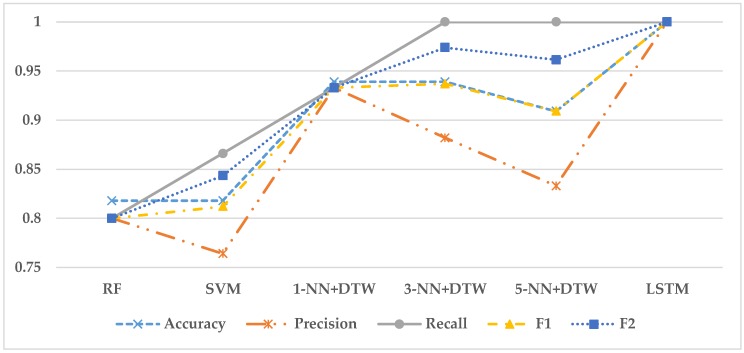
Performance comparison of six models. LSTM shows the best performance in terms of F1 and F2 scores.

To compare the performances among six models, 70:30 holdout technique was used. In this experiment, we had 110 observations. The 70% of the dataset (77 observations) was used for a training set, and the 30% of the dataset (33 observations) was used for a test set. Also, to find the hyperparameters, the 5-fold cross validation method was applied to the training data. In other words, 77 observations of the training set were divided into five folds and then the models trained with four folds are validated with the remained fold to find the best hyperparamters. Finally, the performance of the best model is evaluated with 33 observations of the test set. In this research, accuracy, precision, recall, F1 score, and F2 score were used for the evaluation measures. Table 2 presents the performances of the models based on the five measures, and Figure 6 graphically shows the comparison of their performances.

The results of the experiment showed that the LSTM model was the best classifier in terms of all the measures. The second best model was 3-NN + DTW in terms of F1 and F2 scores (F1 = 0.937 and F2 = 0.974). 1-NN + DTW was the third best model in terms of F1 score, while 5-NN + DTW was the third best model in terms of F2 score. It implies that the 1-NN + DTW model can classify the normal state and the fault state in a balanced way, while the 5-NN + DTW model can predict the fault state more successfully than the normal state.

## 6. Conclusions

In this research, the structure of a fault detection and monitoring system that is essential to smart factories was proposed. In the future, several sensors will be mounted at the facility and an enormous amount of data will be collected from it. To process and analyze these data in a fast and economical way, in the present study, we used the concept of edge computing, which enables the system to perform the necessary tasks within an edge device in a short time. The edge device, which was configured in this study, collects data through sensors, independently processes the data, stores the data in its own database, and analyzes the data with the loaded fault detection model. If the edge device detects any signs of faults, the fault monitor provides a warning sign to the operator for immediate action.

A lightweight real-time fault detection system for edge computing, called LiReD, was proposed in this paper. And, a fault detection model for machine was developed based on LSTM recurrent neural networks. The system collected data in real time generated from facilities using a single-board computer and a sensor. It was implemented and evaluated for an industrial robot manipulator. In the experiment, the LSTM-based fault detection model outperformed five other models such as 1-NN + DTW, 3-NN + DTW, 5-NN + DTW, RF and SVM in terms of five performance measures.

In future work, a more capable single-board computer can be used instead of a Raspberry Pi to create an edge device capable of more complex operations, and various sensors, such as temperature, pressure, and acoustic sensors can be applied to different kinds of facility fault detection. Also, using a simpler algorithm such as GRU, instead of LSTM, may be able to reduce the memory footprint of the proposed system. Moreover, if the fault detection model becomes bigger, the retraining and compression techniques that reduce the volume of the model but increase or maintain its performance can be considered to improve the system. Finally, the fault situation occurs rarely in real production systems, and therefore it is often hard to gather real fault data for the model development. In such situations, one-class classification methods such as Mahalanobis-Taguchi system can also be considered.

## Figures and Tables

**Table 1:** Time domain features.

Feature	Equation	Feature	Equation
Mean $\left(\bar{x}\right)$	$\bar{x}=\frac{1}{n}\sum_{i=1}^{n}x_{i}$	Kurtisus $\left(x_{kur}\right)$	$x_{kur}=\frac{\sum_{i=1}^{n}{\left(x_{i}-\bar{x}\right)}^{4}}{(n-1)x_{std}^{4}}$
Peak $\left(x_{p}\right)$	$x_{p}=\max(x_{i})$	Crest factor (CF)	$\mathrm{CF}=\frac{x_{p}}{x_{rms}}$
Root mean square $\left(x_{rms}\right)$	$x_{rms}=\sqrt{\frac{1}{n}\sum_{i=1}^{n}{x_{i}}^{2}}$	Shape factor (SF)	$\mathrm{SF}=\frac{x_{rms}}{\bar{x}}$
Standard deviation $\left(x_{std}\right)$	$x_{std}=\sqrt{\frac{1}{n-1}\sum_{i=1}^{n}{\left(x_{i}-\bar{x}\right)}^{2}}$	Impulse factor (IF)	$\mathrm{IF}=\frac{x_{p}}{\bar{x}}$
Skewness $\left(x_{ske}\right)$	$x_{ske}=\frac{\sum_{i=1}^{n}{\left(x_{i}-\bar{x}\right)}^{3}}{(n-1)x_{std}^{3}}$	Margin factor (CIF)	$\mathrm{CIF}=\frac{x_{p}}{{\left(\frac{1}{n}\sum_{i=1}^{n}\sqrt{x_{i}}\right)}^{2}}$

**Table 2:** Performance evaluation of six models.

	1-NN + DTW	3-NN + DTW	5-NN + DTW	SVM	RF	LSTM
**Accuracy**	0.939	0.939	0.909	0.818	0.818	1.000
**Precision**	0.933	0.882	0.833	0.764	0.800	1.000
**Recall**	0.933	1.000	1.000	0.866	0.800	1.000
**F1**	0.933	0.937	0.909	0.812	0.800	1.000
**F2**	0.933	0.974	0.961	0.843	0.800	1.000
